# Bevacizumab terminates homeobox B9-induced tumor proliferation by silencing microenvironmental communication

**DOI:** 10.1186/1476-4598-13-102

**Published:** 2014-05-05

**Authors:** Yoshinori Hoshino, Tetsu Hayashida, Akira Hirata, Hidena Takahashi, Naokazu Chiba, Mitsuyo Ohmura, Masatoshi Wakui, Hiromitsu Jinno, Hirotoshi Hasegawa, Shyamala Maheswaran, Makoto Suematsu, Yuko Kitagawa

**Affiliations:** 1 Department of Surgery, School of Medicine, Keio University, 35 Shinanomachi, Shinjuku-ku, Tokyo 160-8582, Japan; 2 Department of Biochemistry, School of Medicine, Keio University, 35 Shinanomachi, Shinjuku-ku, Tokyo 160-8582, Japan; 3 Department of Laboratory Medicine, School of Medicine, Keio University, 35 Shinanomachi, Shinjuku-ku, Tokyo 160-8582, Japan; 4 Massachusetts General Hospital Cancer Center, Harvard Medical School, Bldg 149, 13th Street, Charlestown, MA 02129, USA

**Keywords:** Colorectal cancer, Aangiogenesis, Bevacizumab, Biomarker

## Abstract

**Background:**

Homeobox B9 (HOXB9), a transcriptional factor, regulates developmental processes and tumor progression and has recently been recognized as one of important transcriptional factors related to angiogenesis. This study aimed to investigate the role of HOXB9 in tumorigenesis and angiogenesis.

**Methods:**

We examined the expression of HOXB9 in colorectal cancer using qPCR and in situ hybridization. We also examined the effect of HOXB9 overexpression in colorectal cancer using a proliferation assay, ELISA, a multiplex assay, and xenograft models. The clinical significance of HOXB9 was statistically evaluated in resected specimens.

**Results:**

HOXB9 was expressed in colorectal cancer specimens. HOXB9 induced angiogenesis and tumor proliferation in vitro, which resulted in high tumorigenicity in vivo and poor overall survival. Bevacizumab, an anti-vascular endothelial growth factor (VEGF) antibody, remarkably suppressed tumor proliferation by inhibiting angiogenesis in HOXB9-overexpressing xenografts, and it improved overall survival and provided prolonged progression-free survival in HOXB9-overexpressing patients. A comprehensive multiplex assay of the supernatant of cancer cells co-cultured with human vascular endothelial cells and fibroblasts indicated significantly higher interleukin-6 (IL6) levels than those in the supernatant of monocultured cells. HOXB9 overexpression in clinical specimens was significantly correlated with increased IL6 expression. An IL6-neutralizing antibody inhibited VEGF secretion and tumor proliferation in the co-culture system.

**Conclusions:**

HOXB9 promotes the secretion of angiogenic factors, including VEGF, to induce tumor proliferation through microenvironmental production of cytokines including IL6 signaling. Moreover, silencing of VEGF or IL6 terminates cytokine release in tumor microenvironment. Thus, HOXB9 and IL6 may be potential biomarkers for bevacizumab treatment.

## Background

Homeobox (HOX) genes are key regulators of embryonic development and are evolutionarily conserved [1]. Thirty-nine HOX genes are arranged in 4 different clusters, that is, HOXA–HOXD. Homeobox B9 (HOXB9) is expressed in several adult human tissues, including the endometrium [2], mammary glands [3], and blood precursor cells [4]. HOXB9 has been shown to be overexpressed in human breast cancer and is associated with tumorigenicity, lung metastasis, and radioresistance [5,6]. Especially, HOXB9 induced tumor proliferation and metastasis by activating angiogenesis [5], but little is known about the relationship between HOXB9 expression and angiogenesis in colorectal cancer, which is widely treated by anti-angiogenic therapy in combination with chemotherapy, and about the impact of anti-angiogenic treatment on tumors with angiogenic properties.

Angiogenesis plays critical roles in tumor growth [7,8] and cancer metastasis [9] because the generation of new vessels to meet the metabolic demands of under-perfused regions promotes tumor growth. Vascular network recruitment provides the principal route by which tumor cells exit the primary tumor site and enter the circulation. Thus, angiogenesis has become a target for cancer therapy through inhibition of vascular endothelial growth factor (VEGF) signaling by various drugs, including bevacizumab, sorafenib, and sunitinib.

Bevacizumab is a humanized monoclonal antibody that inhibits VEGF activity. Because VEGF stimulates the angiogenesis of microvessels, bevacizumab is proposed to function by blocking neovascularization in the tumor microenvironment [10,11] and by reducing vascular permeability in tumor microvessels [12,13]. Bevacizumab has become one of the standard components of chemotherapy for metastatic or recurrent colorectal cancer, but no biomarkers are available to determine the efficacy of anti-VEGF therapy.

It is important to investigate the mechanisms underlying anti-angiogenic treatment, especially in highly vascular tumors. Here, the clinical significance of HOXB9 expression was assessed, and the angiogenic and oncogenic functions of HOXB9 were further examined in vivo and in vitro. In the present study, we have demonstrated that anti-angiogenic treatment inhibits tumor proliferation driven by HOXB9 overexpression and decreases the tumor burden by silencing cytokine crosstalk in microenvironment.

## Results

### HOXB9 expression in colorectal cancer patients

**Figure 1:**
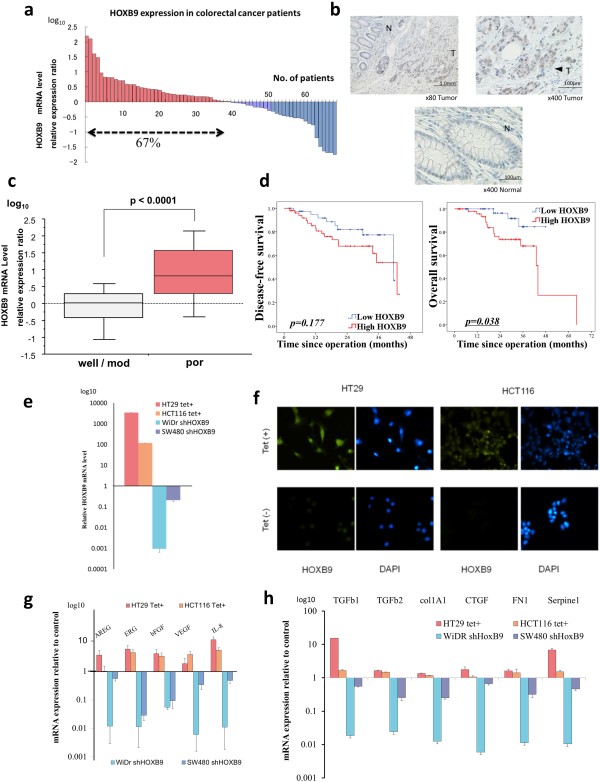
**HOXB9 expression in colorectal cancer. (a)** Waterfall plot of relative mRNA expression levels of HOXB9 in patients at the cancer site compared with those in normal mucosa (n = 69), determined using qRT-PCR in triplicate. **(b)** Immunohistochemical staining of HOXB9 in resected colorectal cancer specimens (T, tumor; N, normal mucosa). **(c)** Relative mRNA expression of HOXB9 in patients according to pathological differentiation (Well/ mod, well or moderately differentiated adenocarcinoma; Por, poorly differentiated adenocarcinoma). **(d)** Kaplan-Meier curves of colorectal cancer patients (n = 93), according to HOXB mRNA expression (Left panel, disease free survival; right panel, overall survival). **(e)** mRNA expression of HOXB9 determined by RT-PCR. **(f)** Immunocytochemistry of HOXB9-overexpressing cell lines. **(g and h)** mRNA expression of angiogenic factors **(g)** and TGF beta family **(h)** in HOXB9-overexpressing cell lines using RT-qPCR in triplicate. Error bars are SDs. P values by the log rank test. Scale bars, 100 μm.

To investigate HOXB9 expression in colorectal cancer, we examined specimens from patients who had p-stage II or III colorectal cancer and had undergone surgery at our institute. HOXB9 mRNA was expressed in approximately 70% of the patients (Figure 1a), and HOXB9 was found to be upregulated in a fraction of tumors, relative to that in the uninvolved adjacent normal mucosa, in both RNA in situ hybridization (Additional file 1: Figure S1a), and immunohistochemical staining (Figure 1b). HOXB9 mRNA expression was significantly higher in poorly differentiated adenocarcinomas than in highly and moderately differentiated types of adenocarcinomas (Figure 1c) and was highly related to liver metastasis (Additional file 1: Figure S1b). Moreover, although HOXB9 did not affect the disease free survival after surgery, increased HOXB9 expression in colorectal cancer was significantly associated with a poor prognosis in overall survival (Figure 1d), as previously reported for breast cancer [14].

### Role of HOXB9 in angiogenesis, TGF-beta axis and tumorigenesis

**Figure 2:**
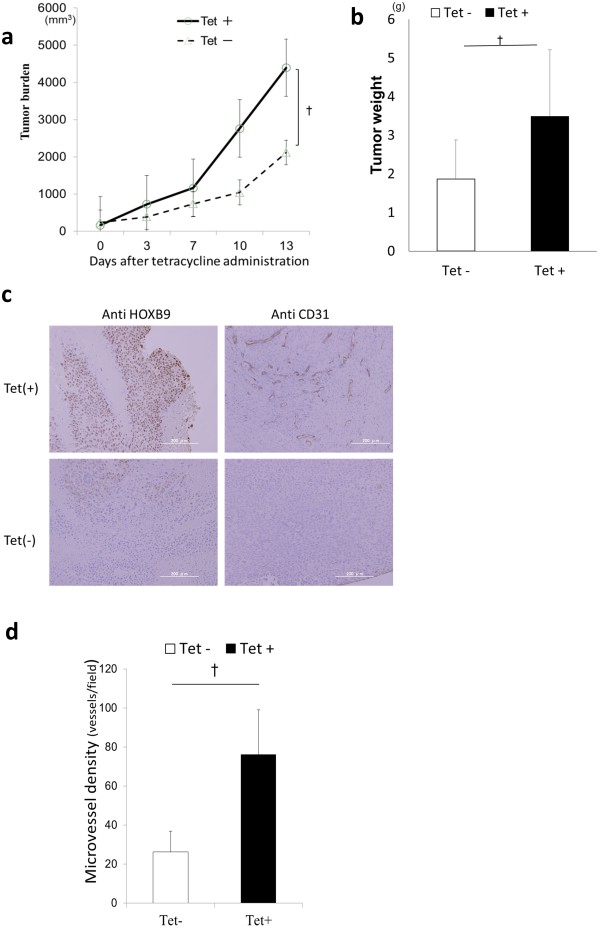
**Impact of HOXB9 expression on tumor xenografts. (a, b)** Subcutaneous xenograft model of HT 29-T cells was established in Balb/c nu/nu mice (n = 8, each). Tumor burden **(a)** and tumor weight **(b)** were evaluated. **(c)** Immunohistochemical staining of paraffin-embedded xenografts obtained from HT29-T cancer cells. Samples were stained in anti-HOXB9 or anti-CD31. **(d)** Microvessel density of xenograft tumor was evaluated and measured in immunohistochemical staining by ant-CD31 antibody in xenografts (n = 8, each). Error bars are SDs. ☆ p < 0.05 and † p < 0.005 by U-test. Scale bars, 200 μm.

We introduced the HOXB9 construct into the HT29 and HCT116 cancer cell lines (HT29-T and HCT116-T, respectively), both of which showed low HOXB9 mRNA expression relative to that in the other human colorectal cancer cell lines (Additional file 1: Figure S1c), in order to evaluate the functional consequence of HOXB9 overexpression in colorectal cancer. HOXB9 overexpression is regulated by a tetracycline repressor (Figure 1e,f and Additional file 1: Figure S1d,e) [15]. Angiogenic factors, including amphiregulin (AREG), epiregulin (ERG), basic fibroblast growth factor (bFGF), interleukin-8 (IL8), and VEGF were significantly upregulated by HOXB9 (Figure 1g). To determine the effect of the loss of HOXB9, we produced short hairpin RNA (shRNA)-expressing HOXB9 lentiviral constructs capable of reducing endogenous HOXB9 expression by up to 99% (Figure 1e). These constructs were used to reduce the expression of angiogenic factors in WiDr and SW480 cell lines (WiDr-S and SW480-S, respectively) that initially had high expression of HOXB9 (Figure 1g). Moreover, the transforming growth factor (TGF) -beta axis was regulated by HOXB9 in colorectal cancer (Figure 1h), similar to the results previously obtained for breast cancer [5]. Next, we investigated the functional significance of these factors in ELISA assays. Significantly increased tumoral secretion of angiogenic factors were confirmed in the supernatants of HOXB9-overexpressing cell lines (Additional file 2: Figure S2a,b). To confirm the role of this acquired angiogenesis of colorectal cancer in vivo, we established human tumor xenografts in BALB/c nude mice. HOXB9-overexpressing xenografts of the HT29-T cell line demonstrated a dramatic increase in tumor burden (Figure 2a,b) and microvessel density (Figure 2c,d and Additional file 2: Figure S2c). The immunohistochemical staining showed higher production of angiogenic factors including IL8 and VEGF in HOXB9 overexpressed tumor (Additional file 2: Figure S2d).

### Inhibition of angiogenesis in HOXB9-overexpressing tumors

**Figure 3:**
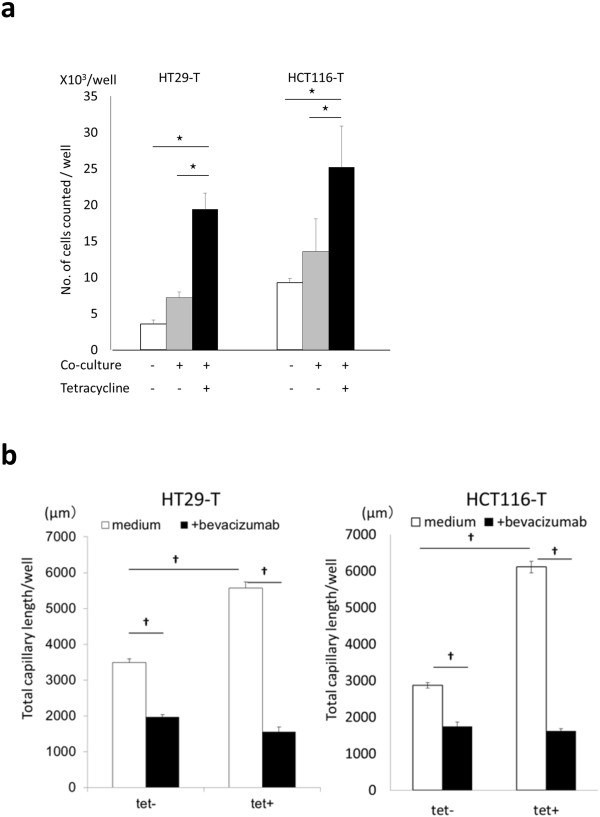
**Impact of cytokine release from HOXB9 overexpressed cells on angiogenesis and cell proliferation. (a)** Cancer cells (3000 cells/well) were seeded on micropore membrane over 24-wells plate and incubated for 24 h before tetracycline administration and/or co-culture with HUVECs and HDFs. After 48 hours incubation with tetracycline and/or co-culture system, the number of cancer cells were counted (n = 8, each). **(b)** Total capillary length of co-cultured HUVECs were evaluated in immunohistochemical staining of CD31 after 48 hours incubation with HOXB9 induced cell lines (n = 8, each). Error bars are SDs. ☆p < 0.05 by the U-test.

The cell proliferation test for 24 hours revealed almost the same proliferation in HOXB9 overexpressed cells as control (Additional file 3: Figure S3a), and the cell viability test for 7 days showed rather smaller survival rate in HOXB9 overexpressed cells in vitro (Additional file 3: Figure S3b). Therefore, the role of the microenvironment in tumor proliferation under the regulation of HOXB9 was investigated, given that the primary difference between the in vivo and in vitro conditions was the existence of a microenvironment including vessels and fibroblasts. When HUVECs and human dermal fibroblasts (HDFs) were co-cultured with cancer cell lines to mimic the microenvironment (Additional file 3: Figure S3c,d), HOXB9 significantly promoted cell proliferation (Figure 3a). Then, we hypothesized that anti-angiogenic treatment may cause strong regression in tumors with such angiogenic properties as HOXB9, which depends tumor proliferation on enhanced angiogenesis and stromal reaction in the microenvironment. In order to evaluate the impact of anti-angiogenic agents on tumor microenvironment in vitro, the tube formation of HUVECs co-cultured with HOXB9-regulated cancer cells was estimated under administration of bevacizumab. HOXB9 significantly enhanced angiogenesis, and bevacizumab administration significantly reduced angiogenesis of HUVECs co-cultured with HOXB9-overexpressing cancer cells (Figure 3b and Additional file 3: Figure S3e,f).

**Figure 4:**
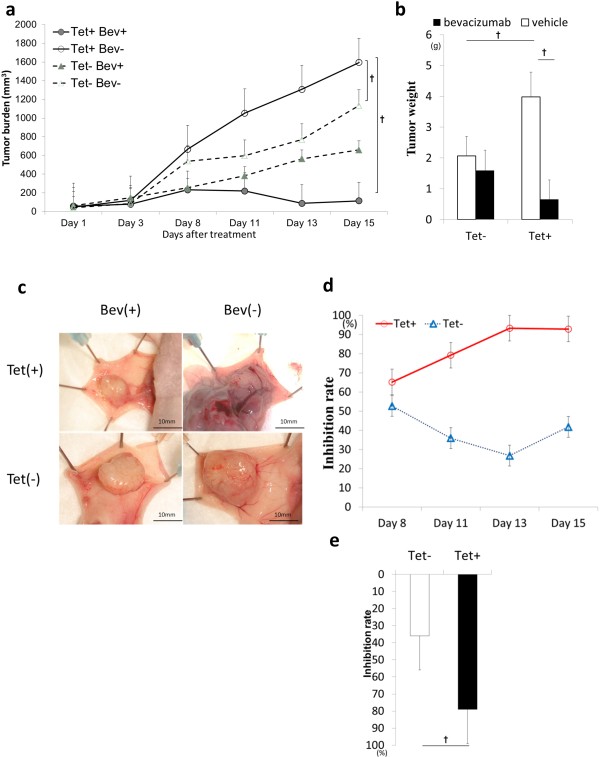
**Bevacizumab treatment and HOXB9 expression.** Subcutaneous xenografts of HT 29-T cells in Balb/c nu/nu mice were treated with bevacizumab (n = 8, each). Tumor growth under bevacizumab treatment were evaluated in tumor burden **(a)**, tumor weight **(b)**, tumor specimen **(c)**, and tumor inhibition rate **(d)**. **(e)** Tumor inhibition rate was calculated in HT 29-T on day 13. Error bars are SDs. ☆p < 0.05 and † p < 0.005 by U-test.

Next, BALB/c nude mice transplanted with xenografts that had been previously established using HOXB9-overexpressing colon cancer cells were treated intraperitoneally with bevacizumab (5 mg/kg, weekly). Both of HOXB9-induced cancer cells showed accelerated tumorigenesis. However, the bevacizumab treatment dramatically reduced tumor volume and tumor weight both in HT29-T cells (Figure 4a,b) and in HCT116-T (Additional file 4: Figure S4a,b). Tumor gross vascularity was also reduced by bevecizumab (Figure 4c). The growth inhibition mediated by bevacizumab significantly increased in HOXB9-overexpressing tumors (Figure 4d,e and Additional file 4: Figure S4c,d). We further established an intrasplenic injection (ISP) model of HCT116-T cells in BALB/c nude mice to determine whether anti-angiogenic treatment with bevacizumab prolongs survival in angiogenic tumors. HOXB9 overexpression was associated with longer third-quadrant survival upon bevacizumab treatment (Additional file 5: Figure S5a,b). To vertify our hypothesis that the loss of angiogenic factors plays an important role in tumor shrinkage in angiogenesis-driven tumor, sunitinib malate, which is a multiple tyrosine kinase inhibitor with effects including VEGF inhibition, was also administered in HOXB9 overexpressed tumor. Sunitinib also suppressed cell proliferation in HOXB9-overexpressing breast cancer xenografts (Additional file 5: Figure S5c).

### Enhanced cytokine release from microenvironment

**Figure 5:**
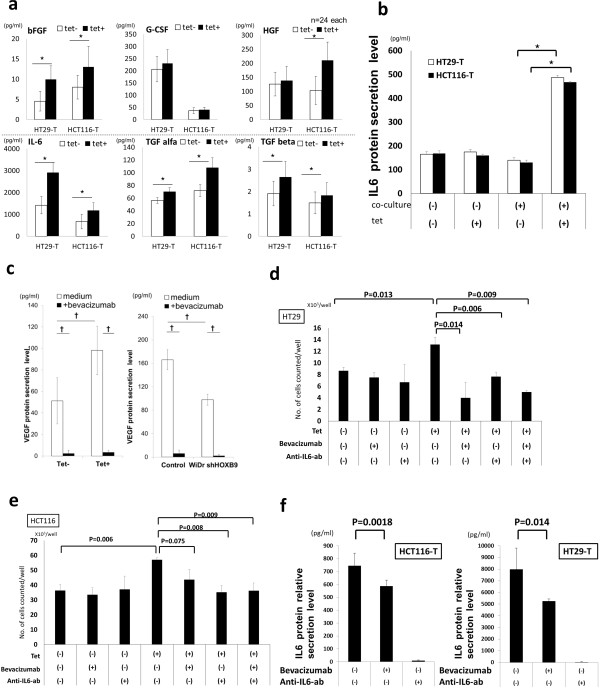
**HOXB9 expression and co-culture systems. (a)** Cancer cells were co-cultured with HUVECs and human fibroblasts for 48 hours. Cytokines in supernatants were evaluated by multiplex assays (n = 24, each). **(b)** IL6 secretion levels in supernatants were compared by ELISA between the monoculture and co-culture systems in HT29-T or HCT116-T cancer cells. HOXB9 overexpression was induced by tetracycline (1 μg/ml). **(c)** VEGF secretion and bevacizumab treatment in the co-culture system, detected by ELISA (HT 29-T, left panel; WiDr-S right panel). **(d, e)** Tumor proliferation of HT29-T **(d)** and HCT116-T **(e)** cell lines on treatment with bevacizumab and/or an IL6-neutralizing antibody in the co-culture system with HUVECs and human fibroblasts. **(f)** IL6 secretion under treatment with bevacizumab and/or IL6 neutralizing antibody in the co-culture system with HUVECs and human fibroblasts. Error bars are SDs. ☆ p < 0.05 and † p < 0.005 by the U-test.

To determine the key factors for enhanced tumor growth in microenvironment, we performed comprehensive multiplex assays involving the co-culture supernatants and observed significant elevation of the levels of several cytokines, including interleukin-6 (IL6) (Figure 5a). We then focused on the differences between the secretion of IL6 in monoculture and co-culture systems to elucidate the impact of microenvironment on tumor proliferation. The concentration of IL6 was dramatically higher in the co-culture system than in the mono-cultures (Figure 5b and Additional file 6: Figure S6a), suggesting enhanced IL6 secretion in microenvironment including tumor and stromal cells. To confirm the importance of microenvironmental IL6 secretion, cell proliferation in the presence of an IL6 neutralizing antibody was evaluated. We also used bevacizumab to inhibit the activity of VEGF released from cancer cells (Figure 5c). Both the IL6 neutralizing antibody and bevacizumab produced significant reduction of cancer cell proliferation (Figure 5d,e and Additional file 6: Figure S6b). Moreover, IL6 secretion in the supernatant dramatically decreased on administration of bevacizumab in co-culture systems (Figure 5f).

### Clinical significance of HOXB9 on bevacizumab treatment

**Figure 6:**
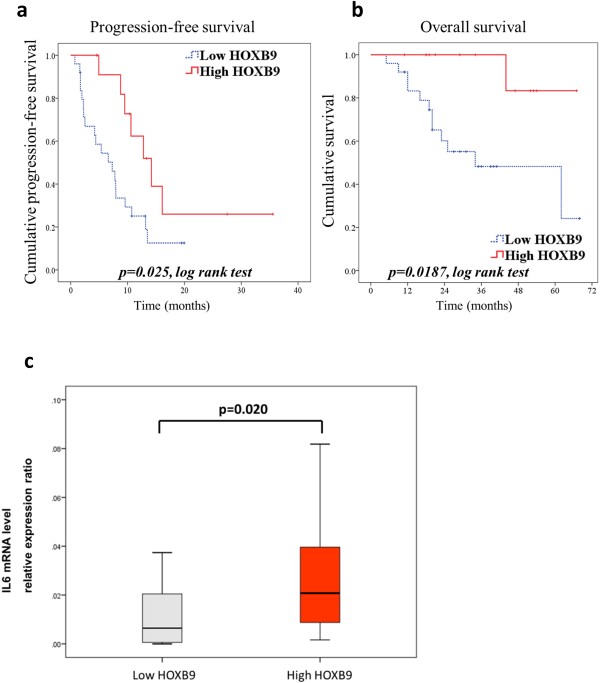
**Clinical data for colorectal cancer patients undergoing bevacizumab treatment.** HOXB9 mRNA expression levels were evaluated in triplicate by qRT-PCR in patients who showed recurrence after surgery and were treated by chemotherapy combined with bevacizumab **(a and b)** Kaplan-Meier curves of colorectal cancer patients, according to HOXB mRNA expression. Progression-free survival **(a)** and overall survival **(b)**. p values by the log rank test. **(c)** IL6 mRNA expression in colorectal cancer specimens. Error bars are SDs. P values by the U-test and log rank test.

To clarify the clinical significance of anti-angiogenic treatment for angiogenic colorectal cancer, we conducted a retrospective study of patients who had undergone surgery between 2001 and 2010 at our institute and were treated by chemotherapy, including bevacizumab (n = 39). Specimens obtained from patients before chemotherapy were analyzed by Quantitative reverse transcription-PCR (qRT-PCR). The demographic and clinical data showed no significant differences with regard to age, sex, pathological stage, or chemotherapy between groups with high (n = 13) or low (n = 26) expression of HOXB9 (Additional file 7: Table S1). High expression of HOXB9 mRNA was significantly associated with longer overall survival and progression-free survival after treatment with bevacizumab combined with chemotherapy (Figure 6a,b), and mRNA expression of HOXB9 was significantly correlated with IL6 mRNA expression in clinical specimen (Figure 6c).

## Discussion

HOXB9 has been reported to be overexpressed in neoplastic tissues [16]; to promote the expression of angiogenic factors, ErbB, and TGF-beta ligands; and to promote angiogenesis and distant metastasis in breast cancer [5]. From a biological perspective, multiple HOX-binding sites have already been identified in the promoters of angiopoietin-like 2, IL8, TGF-beta2, VEGF, and bFGF [17]. AREG, ERG, VEGF, and bFGF were previously revealed to be the direct targets of HOXB9 [5,18]. In bioinformatic analysis using PROMO3.0 (http://alggen.lsi.upc.es/cgi-bin/promo_v3/promo/promoinit.cgi?dirDB=TF_8.3), we found multiple sequences in the promoter regions of IL8 where HOX genes were predicted to bind*.* In the present study, we demonstrated that HOXB9 also promoted the expression of angiogenic factors and TGF-beta ligands in colorectal cancer. Tumorigenesis was enhanced by the microenvironment, as demonstrated by stromal interaction and neovascularization in HOXB9-overexpressing tumors in vivo. In addition to angiogenesis, the stromal response has been suggested as one of the determinants of tumor proliferation and recurrence in colorectal cancer [19]. The tumor stroma is activated by TGF-beta signaling, which leads to upregulation of extracellular matrix proteins and inflammatory, angiogenic, and invasion factors that promote the growth of tumor cells [20,21]. A study involving chromatin immunoprecipitation previously demonstrated that HOXB9 binds to the TGF-beta2 promoter region as a transcriptional factor to regulate gene expression [5]. Taken together, the activation of angiogenesis and TGF-beta axis in vitro may result in strong tumorigenesis in vivo and poor overall survival in colorectal cancer patients.

To vertify our hypothesis that angiogenic tumors which depend the tumorigenity on angiogenesis are more sensitive to anti-angiogenic treatment, the present study has demonstrated three important findings: the relationship of HOXB9 to colorectal cancer tumorigenicity in vitro and in vivo in terms of angiogenesis and TGF beta signaling; the association of VEGF and anti-angiogenic agents with the tumorigenic HOXB9 effect; and, the association of HOXB9 with accelerated cytokine release from tumor microenvironment. For the investigation of tumor suppression by bevacizumab administration, we performed the multiplex assay for comprehensive detection of enhanced cytokine production induced by HOXB9 in co-culture experiments. Our study which compared co-culture with mono-culture demonstrated that tumorigenic effects of HOXB9 in the microenvironment resulted in the enhanced release of various cytokines and that IL6 release from tumor microenvironment plays an important role in tumor proliferation. Not only silencing of IL6 led to the reduction in the proliferation of tumor in co-culture experiments, but also bevacizumab administration reduced IL6 release itself from tumor microenvironment. Thus, these on-and-off results for HOXB9 expression and neutralizing antibodies suggest our hypothesis that bevacizumab inhibits HOXB9 induced tumor proliferation by silencing microenvironmental cytokine release including VEGF and IL6 (Additional file 6: Figure S6c). The microenvironment in humans includes not only vessels and fibroblasts but also immune systems, which results in complicated interactions. In the present study, the predominance of cytokine secretion between tumor and stroma is not evident, and a role for IL6 as another mediator cannot be excluded. But our data suggest a possible role for microenvironmental IL6 in tumor cell proliferation, at least with regard to angiogenesis.

Our results are consistent with those of previous studies demonstrating that IL6 affects microenvironment. IL6 levels are known to be increased in most epithelial tumors [22], and high serum IL6 levels are associated with poor clinical outcomes in patients with colorectal cancer [23] or ovarian cancer [24]. IL6 is a key mediator in a mouse model of microbially induced colorectal cancer [25], possibly through induction of cancer-related molecular pathways such as those involving STAT3 [26].

Several limitations affect the interpretation of the present findings. First, this translational study was based on a retrospective analysis in a single center and a small number of patients were included. Second, the selection of patients for the bevacizumab treatment was subjective. The clinical significance of HOXB9 and IL6 must therefore be addressed in a prospectively planned multicenter trial. Multigene assays involving a large number of specimens may provide more reliable insights into tumor biology and the response to bevacizumab regimens. Third, the relationship between the immunological response and chemotherapy combined with anti-angiogenic treatment is unclear because bevacizumab was not administered as a single agent in our clinical study. Fourth, the impact of a combined cytotoxic chemotherapy regimen is unclear; the results obtained in the present study are biased by the chemotherapy itself.

## Conclusions

HOXB9 is also expressed in colorectal cancer and functions as a strong tumorigenic factor through activation of the TGF-beta axis and angiogenesis. Colorectal cancer patients with HOXB9 overexpression had poor overall survival in this study, but conversely demonstrated better overall survival with bevacizumab treatment. Bevacizumab suppressed secretion of IL6 into the microenvironment in vitro, which suggests a possible mechanism for the increased response to bevacizumab treatment. Previous studies have reported the clinical impact of some genes, including VEGF splice isoforms or neuropilin-1 (NRP-1) [27]. However, to date, no biomarkers have been identified in randomized controlled trials on the clinical response of anti-angiogenic treatments in colorectal cancer [28]. Further prospective studies on HOXB9 and IL6 in different specimens such as serum samples are important to clarify the utility of these molecules as biomarkers.

## Methods

### mRNA expression

Our analysis included colorectal tumors of patients who presented between January 2004 and January 2008 at Keio University Hospital; these data were obtained from the hospital colorectal cancer database. Among the samples of consecutive sporadic colorectal cancer patients who received a diagnosis of p-stages II or III disease, 69 panels of frozen tissue specimens containing cancerous and matched normal mucosal tissue were available for qRT-PCR assessment. All samples were obtained from surgically resected material, immediately frozen in liquid nitrogen, and stored at −80°C. Adequate lymph node dissection was performed in all these cases, and a pathological diagnosis was made according to the TNM classification. HOXB9 mRNA expression was examined at cancer sites and in normal mucosa in consecutive pStages II and III patients who had undergone surgery at our institute during 2004–2008. The expression ratios (cancer site to normal mucosa) were examined using qRT-PCR. RNA was extracted from the cells by using the RNeasy kit (QIAGEN, Valencia, CA). Conditions for semiquantitative amplification of cDNA were 95°C for 2 min, followed by 25 cycles of 95°C for 30 s, 56°C for 30 s, and 72°C for 60 s, with a final extension cycle of 72°C for 10 min. RT-PCR analysis was run in triplicate for each sample on a Light Cycler 480 Real-Time PCR System using SYBR Green 1 Master Mix (Roche). The following program was run: preincubation for 5 min at 95°C, amplification for 45 cycles (10 s of denaturation at 95°C, 10 s of annealing at 57°C, and a 10-s extension at 72°C), and melt-curve analysis. mRNA levels of the target gene were normalized against mRNA levels of GAPDH, which was used as an internal control. The sequences of all the primers used are listed in Additional file 8: Table S2. Written informed consent was taken and the study was approved by the ethics committee of the School of Medicine of Keio University.

### In situ hybridization

The HOXB9 probe for ISH was generated as described previously [5]. Briefly, the plasmid encoding HOXB9 was cleaved with Hinc II and XmaI (New England Biolabs). The acquired 286-bp fragment was outside the homeodomain and spanned nucleotides 26–324 of the HOXB9 transcript. This region had 100% homology to HOXB9, as expected, and had little homology with other members of the HOX family or other HOX genes. After linearization of the plasmid, in vitro transcription was carried out, using 11-digoxogem-UTP (Roche) and 2 units/μL of either T3 of T7 RNA polymerase (Promega) for sense and antisense probes, respectively. Riboprobes were purified by ethanol precipitation, and their size and integrity checked by gel electrophoresis. The tissues were dehydrated, washed twice with PBS, and treated with 3% hydrogen peroxide for 15 min. Following treatment with proteinase K (10 μg/ml) for 9 min, tissues were washed with PBS and refixed with 4% paraformaldehyde in PBS for 5 min. The tissues were pre-hybridized for 1 hr at 65°C before hybridizing with sense or anti-sense digoxigenin-labeled riboprobes overnight at 70°C. After hybridization, samples were placed in 1% sheep serum for 30 min at room temperature, and then incubated with anti-digoxygenin-AP (Roche) antibody for 2 hours at room temperature. BM-Purple precipitation (Roche) was used to detect the signal.

### Cell lines and cell culture

The human colorectal cancer cell lines SW480, WiDr, HT-29, and HCT116 were obtained from the American Type Culture Collection (Rockville, MD). The cell lines HT29 and HCT116 were seeded at a density of 1 × 10^6^/ml in RPMI 1640 medium (Gibco, MA), and SW480 and WiDr cells were seeded a density of 1 × 10^6^/ml in Dulbecco’s modified Eagle medium. Both media were supplemented with 10% FBS (CSL Ltd., Australia), 100 IU/ml penicillin, and 50 mg/ml streptomycin sulfate. HT29 and HCT116 cells with low HOXB9 expression were infected with HOXB9-expressing lentiviral constructs (Invitrogen). For the Tet-regulated system, the entry vector was transferred to the lentiviral pLenti4/TO/V5-DEST vector and used in combination with the Tet-repressor vector pLenti6/TR (Invitrogen) according to the manufacturer’s instructions. The induction of HOXB9 was regulated by the addition of 1 μg/ml tetracycline (Invitrogen). To knock down HOXB9 expression, viral constructs of shRNA were generated, and SW480 and WiDr cells were infected as described previously [5].

### Edu cell proliferation assay

DNA synthesis was measured using 5-ethynyl-2´-deoxyuridine (EdU) Flow Cytometry Assay Kits (Life Technologies, Tokyo, Japan). Experiments were performed according to the manufacturer’s instructions. Briefly, 3 × 10^6^ cells were seeded into 10 cm dish and incubated for 24 hours at 37°C with or without 1 μg/ml tetracycline (Invitrogen). Cells were incubated for 2 hours with 20 μM EdU. Subsequently, cells were processed using the Click-iT EdU Flow Cytometry kit. EdU incorporation was measured by Flow cytometer (Gallios Flow Cytometer, Beckman Coulter).

### MTT cell proliferation assay

Cell proliferation was measured using the 2,3-bis (2-methoxy-4-nitro-5-sulfophenyl)-2H-tetrazolium-5-carboxanilide inner salt (MTT) assay. Briefly, 5,000 cells per well in 96-well microtiter plates (Sumilon, Sumitomo Bakelite Co., Tokyo, Japan) were incubated for 24 h, followed by continuous exposure to 1 μg/ml tetracycline (Invitrogen) for 6 days. The absorbance in the wells was measured at every 2 days using a NJ-2300 microplate spectrophotometer at 540 and 630 nm (Immuno Reader, Nalge Nunc International, Rochester, NY).

### Western Blotting, Immunocytochemistry, and immunohistochemistry

Expression of proteins were detected in western blotting using antibody against HOXB9 (Santa Cruz Biotechonlogies), pAKT (Cell Signaling Technology), AKT (Cell Signaling Technology), VEGF (ABCAM) and GAPDH (ABCAM). The western blot protocols used have been described previously [29]. For immunocytochemistry, cells were plated in 8-well chamber slides (Lab Tek) and treated as described previously [5]. Briefly, after blocking, the cells were incubated with an anti-human HOXB9-specific antibody (rabbit polyclonal antibody; Santa Cruz Biotechnology, Santa Cruz, CA) at a dilution of 1:250 or an anti-CD31 antibody (ab28364; ABCAM) at 1:200 for 1 h at room temperature in incubation buffer (phosphate-buffered saline (PBS) containing 1% BSA). For immunohistochemistry, HOXB9, CD31, CD34, IL8, and VEGF expression was detected using the anti-human HOXB9-specific antibody at a dilution of 1:250 (Santa Cruz, CA), the anti-CD31 antibody at a dilution of 1:200 (ab28364; ABCAM), the anti-CD34 antibody at a dilution of 1:200 (ab8158; ABCAM), anti-IL8 antibody at a dilution of 1:1000 (ab7747; ABCAM), the anti-VEGF antibody at a dilution of 1:1000 (ab46154; ABCAM).

### Angiogenesis in vitro and co-culture systems

Angiogenesis was evaluated based on the microvessel density of HUVEC cultures. Culture supernatants without FBS or antibiotic supplementation were assessed using an angiogenesis kit (Kurabo, Okayama, Japan). Experiments were performed according to the manufacturer’s instructions. The angiogenesis kit consisted of a 24-well cluster dish in which HUVECs and fibroblasts were seeded under optimal conditions for capillary tube formation. The microvessel density was calculated as the sum of the total capillary length in 6 areas at 200× magnification by using the ImageJ software (NIH, Bethesda, MD). HUVECs and fibroblasts were co-cultured with HOXB9-overexpressing cancer cells and separated from the cancer cells by using micropore membranes. Each well was cultured with serum-free medium corresponding to each cell line, respectively.

Tetracycline was administered to regulate HOXB9 induction after 24 hours of co-culture. Endogenous IL6 was neutralized by application of an anti-IL6 antibody, and VEGF neutralization was assayed in a co-culture system in which 5 × 10^3^ HOXB9-overexpressing cancer cells were seeded per well in a medium supplemented with 1 μg/ml anti-human IL6 antibody (R&D Systems) and/or 1 μg/ml bevacizumab. HOXB9 expression was controlled by the addition of tetracycline.

### Multiplex assay

Cytokine levels in cell culture supernatants were quantified using ELISA (BD Biosciences Pharmingen for VEGF, HGF, CXCR8, and IL6). Comprehensive proliferative cytokine levels in co- and monoculture supernatants were measured using a Procarta Cytokine Profiling kit (Affymetrix, Santa Clara, CA, USA), which utilizes xMAP multianalyte profiling Luminex technology for simultaneous detection and quantification of multiple protein targets. Fluorescently encoded antibody beads were detected individually in a flow cytometer, and 9 cytokines (bFGF, G-CSF, GM-CSF, HGF, IL1beta, IL6, MCSF, TGFbeta, and TGFalpha) were evaluated.

### Xenograft experiments

All the animals were cared for and experiments were performed according to AAALAS guidelines using protocols approved by the institutional review board and the institutional animal care and use committee of Keio University or Massachusetts General Hospital. Six-week-old Balb/c female nude mice were subcutaneously implanted with 4 × 10^6^ cells in 100 μl of PBS (n = 8). If the tumor size was greater than 75 mm^3^, oral tetracycline administration was started on day 1. Tetracycline (Sigma-Aldrich) was dissolved in distilled water at a concentration of 1 mg/ml. Bevacizumab (5 mg/kg) or PBS was intraperitoneally administered weekly. The tumor volume was calculated from the pi-based ellipsoid volume formula π/6 × length × width × height. Growth inhibition of the tumor was calculated as (1 – (mean tumor volume of treated group/mean tumor volume of vehicle group)) × 100. An intrasplenic injection (ISP) model was established to create liver metastasis as described previously [30,31]. Briefly, nude mice were anesthetized by ether inhalation, and open laparotomy was performed. HOXB9-overexpressing cancer cells (5 × 10^6^ cells of HCT116-T in 100 μl of PBS) were injected into the spleen, and splenectomy was then performed. Tumor samples were immunostained using an anti-CD31 antibody (Abcam, Cambridge, UK) and then counterstained with DAPI. Vessels were counted using a microscope in areas of the tumor containing the highest number of capillaries and small venules. Highly vascular areas were first identified by scanning tumor sections, and the vessels were counted in 6 such areas at 200× magnification. Bevacizumab was purchased from Chugai Pharmaceutical Co. Ltd (Tokyo, Japan). MCF10A cells expressing activated G12V H-RasV12-expressing lentivirus and either LacZ or HOXB9 were generated using standard protocols. Xenografts were established by injecting 5 × 10^6^ cells in 100 μl of PBS into the mammary fat pad of 6-week-old female Swiss nu/nu mice. Tumor volumes were measured at regular intervals.

Sunitinib (SU11248; Sutent) was purchased from LC Laboratories. MCF10A cells expressing activated G12V H-Ras-expressing lentivirus and either LacZ or HOXB9 were injected into the mammary fat pads of mice (n = 16). After 2 weeks of tumor growth, 8 animals from each group were treated with 40 mg/kg sunitinib orally every day for up to 14 days. Sunitinib was dissolved in 0.5% carboxymethyl cellulose. The remaining 8 animals from each group were treated with the vehicle.

### Patients and samples

Our analysis included 456 colorectal tumors of patients who presented between January 2006 and January 2008 at Keio University Hospital; these data were obtained from the hospital colorectal cancer database. Patients who showed recurrence after surgery and were treated by chemotherapy combined with bevacizumab were selected from the database. Among the specimens from 77 consecutive patients, 39 specimens were available and were analyzed by qRT-PCR. The study was approved by the ethics committee of the School of Medicine of Keio University.

### Statistical analysis

Statistical analysis was performed using the SPSS 18.0 statistical software (SPSS, Chicago, IL). Analyses of age, pathologic tumor size and weight, mRNA expressions, and total capillary length were performed using the Mann–Whitney U test. The tumor size, weight, and burden were statistically evaluated at the time of sacrifice in xenograft models. Disease-free survival (DFS) and overall survival (OS) curves were drawn according to Kaplan–Meier estimates and compared by log-rank tests. DFS was defined as the absence of local or regional recurrence, distant metastasis, second primary carcinoma, or death from any cause. The clinicopathologic categorical variables were compared using Fisher’s exact test or logistic regression where appropriate. The cutoff value for HOXB9 mRNA expression was determined according to the receiver operating characteristic curve. P < 0.05 was defined as significant.

## Abbreviations

HOXB9: Homeobox B9; HOX: Homeobox; IL6: Interleukin-6; VEGF: Vascular endothelial growth factor; MTT: 2,3-bis (2-methoxy-4-nitro-5-sulfophenyl)-2H-tetrazolium-5-carboxanilide inner salt; EdU: 5-ethynyl-2´-deoxyuridine; AREG: Amphiregulin; ERG: Epiregulin; bFGF: basic fibroblast growth factor; IL8: Interleukin-8; ISP: Intrasplenic injection; DFS: Disease-free survival; OS: Overall survival; shRNA: short hairpin RNA; qRT-PCR: quantitative reverse transcription- polymerase chain reaction; TGF: Transforming growth factor.

## Competing interests

Yuko Kitagawa (Keio University) received research support from Chugai Pharmaceutical Co Ltd. The remaining authors disclose no conflicts.

## Authors’ contributions

TH, MO, MW, HJ, and HH designed the experiments. YH and HA conducted the majority of the experiments. NC and HS performed mouse experiments. MO and MW performed protein assays. HT performed EdU assays. TH, MS, and YK conceived the project and participated in experimental design. YH wrote the manuscript. TH, MO, and MW participated in writing the manuscript. All the authors have discussed the results and commented on the manuscript.

## Authors’ information

Y.H., T.H., H.T., A.H., N.C., and M.O. are assistant professors of Keio University, School of Medicine. M.W. and H.J. are instructors of Keio University, School of Medicine. H.H. is an associated professor of Keio University, School of Medicine. M.S. is an associate professor of Harvard Medical School. M.S. and Y.K. are professors of Keio University, School of Medicine.

## Supplementary Material

Additional file 1: Figure S1 HOXB9 expression in colorectal cancer is associated with poor prognosis and enhanced angiogenesis. Relative mRNA expression levels HOXB9 in patients at the cancer site compared with those in normal mucosa, determined using qRT-PCR. **(a)** In situ hybridization HOXB9 in resected colorectalcancer specimens (As, AntiSense). **(b)** HOXB9 expression of resected specimens and recurrence. **(c)** mRNA expression of HOXB9 in human colorectal cancer cell lines were evaluated in triplicate in qRT-PCR **(d)** mRNA expression of HOXB9 determined by RT-PCR. **(e)** Western blotting of HOXB9 and related proteins. p values by the U test. Click here for file

Additional file 2: Figure S2 **(a, b)** Impact of HOXB9 expression on cytokine and cell proliferation in vitro. Cytokine secretion in HOXB9-over expressing cell lines (n=8). CXCR8 **(a)** and VEGF **(b)**. **(c)** Immunohistochemical staining of paraffin-embeddedxenografts obtain from HT29-T cancer cells. Samples were stained in anti-HOXB9 or anti-CD34 antibody. **(d)** Immunohistostaining of xenografts using anti IL8 antibody and anti VEGF antibody. Error bars are SDs. * p <0.05 by U-test. Click here for file

Additional file 3: Figure S3 **(a and b)** In vitro cell proliferation of HOXB9 regulated cells evaluated in Edu assays **(a)** and MU assays **(b)**. **(a)** Cell proliferation for 24 hours after the induction of HOXB9 in HT29-T cells and HCT116-T cells were evaluated in flow cytometry. **(b)** Cell proliferation and survival for 7 days in four HOXB9 regulated cell lines were examined in MU assays (n=8 each). **(c and d)** Schematic illustration of coculture systems of tumor cells and HUVECs co-culture systems. Tumors were co-cultured with HUVECs and human fibroblast in the same medium via micro-pore membrane with or without tetracycline to regulate HOXB9 expression to mimic the microenvironment **(c)**. Tumors communicated with microenvironmental cells in a paracrine way **(d)**. **(e and f)** Immunocytochemical stainings of HUVECs in co-culture system with or without bevacizumab. HUVECs co-cultured with HT29-T **(e)** or WiDr-S **(f)** were stained using anti CD31 antibody. Error bars are SDs. Click here for file

Additional file 4: Figure S4 Bevacizumab treatment and HOXB9 expression **(a-d)** Tumor growth under bevacizumab treatment in HCT 116-T xenograft models in Balb/c flu/flu mice (n = 8 each). **(a)** Tumor burden were calculated every 3-4 days. **(b)** Tumor weight were measured at the time of sacrifice. **(c)** The size of specimens were also evaluated at the time of sacrifice. **(d)** Tumor inhibition rate was calculated in Ha 116- T on day 21. Error bars are SDs. *p < 0.05 and tp < 0.005 by U-test. Click here for file

Additional file 5: Figure S5 Xenografted animals treated with bevacizumab and sunitinib. **(a and b)** HOXB9 expression and bevacizumab treatment were evaluated in ISP model of HCT116-T cells in Balb/c mice (n = 8). Kaplan-Meier curves for each group **(a)** and third-quadrant survival period for bevacizumab treatment **(b)** were analyzed in these mice. **(c)** Tumor growth under sunitinib treatment in an MCF 1OA-RAS xenograft model in Balb/c nu/nu mice (n = 8). Error bars are SDs. tp < 0.005 by U-test. Click here for file

Additional file 6: Figure S6 HOXB9 expression and co-culture systems in WiDr cells. **(a)** Cytokines in supernatants of co-cultured cell lines, detected by ELISA (no significant difference, n8 each). **(b)** Tumor proliferation of WiDr shHOXB9 and scramble cell lines on treatment with bevacizumab andlor an 1L6-neutralizing antibody in the co-culture system. **(c)** Scheme of the hypothesis in the present study. Error bars are SDs. p values by U-test. Click here for file

Additional file 7: Table S1 Consecutive colorectal cancer patients who received bevacizumab treatment after tumor resection were enrolled in this study. Click here for file

Additional file 8: Table S2 Primer sequence in this study. Click here for file
