# Supplementary figures and images for: Bevacizumab terminates homeobox B9-induced tumor proliferation by silencing microenvironmental communication
Source: Mol Cancer. 2014 May 5;13:102. doi: 10.1186/1476-4598-13-102 (PMC4023179; doi:10.1186/1476-4598-13-102)

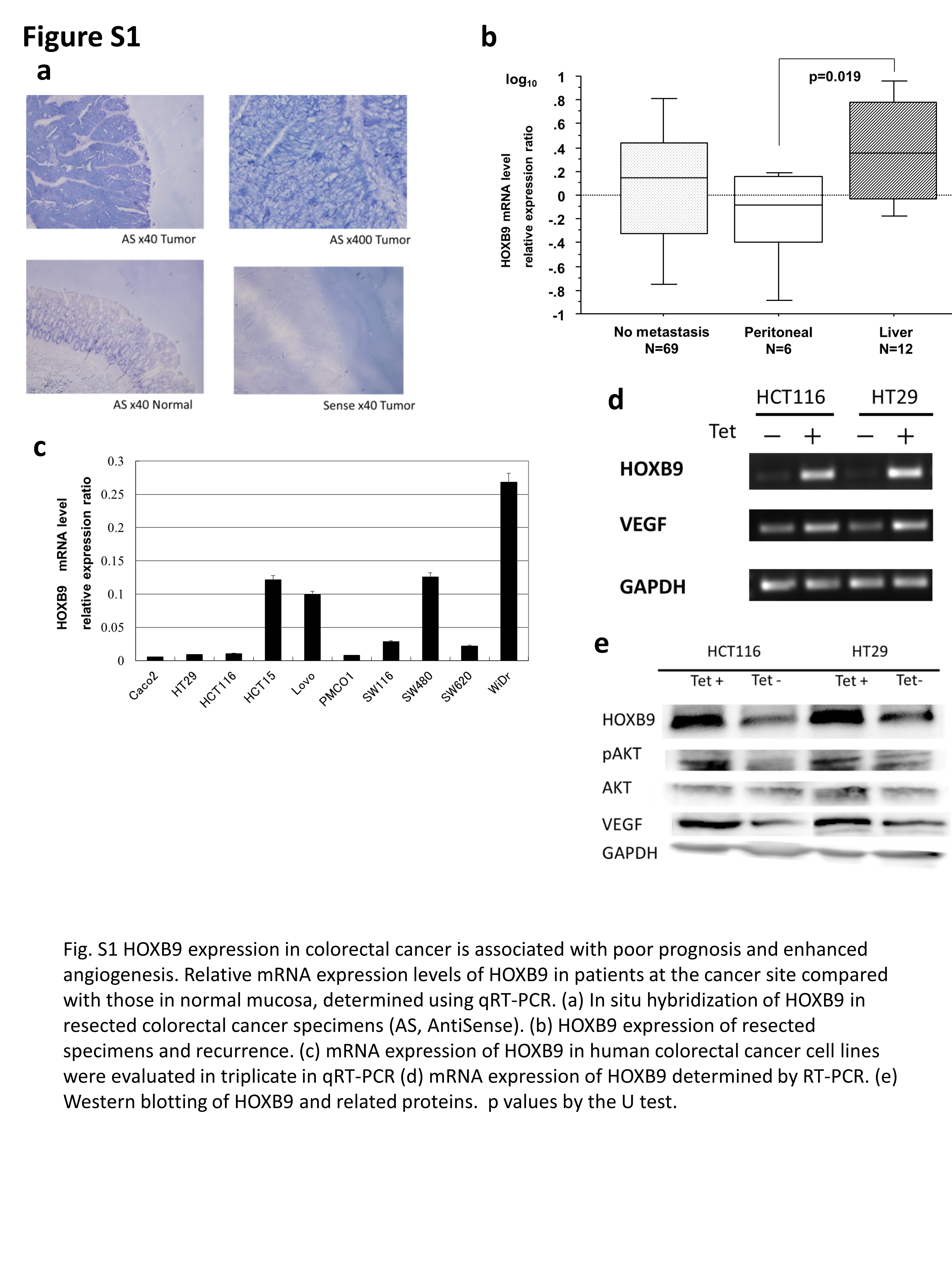

Supplement: Additional file 1: Figure S1 — HOXB9 expression in colorectal cancer is associated with poor prognosis and enhanced angiogenesis. Relative mRNA expression levels HOXB9 in patients at the cancer site compared with those in normal mucosa, determined using qRT-PCR. (a) In situ hybridization HOXB9 in resected colorectalcancer specimens (As, AntiSense). (b) HOXB9 expression of resected specimens and recurrence. (c) mRNA expression of HOXB9 in human colorectal cancer cell lines were evaluated in triplicate in qRT-PCR (d) mRNA expression of HOXB9 determined by RT-PCR. (e) Western blotting of HOXB9 and related proteins. p values by the U test. [file 1476-4598-13-102-S1.tiff]

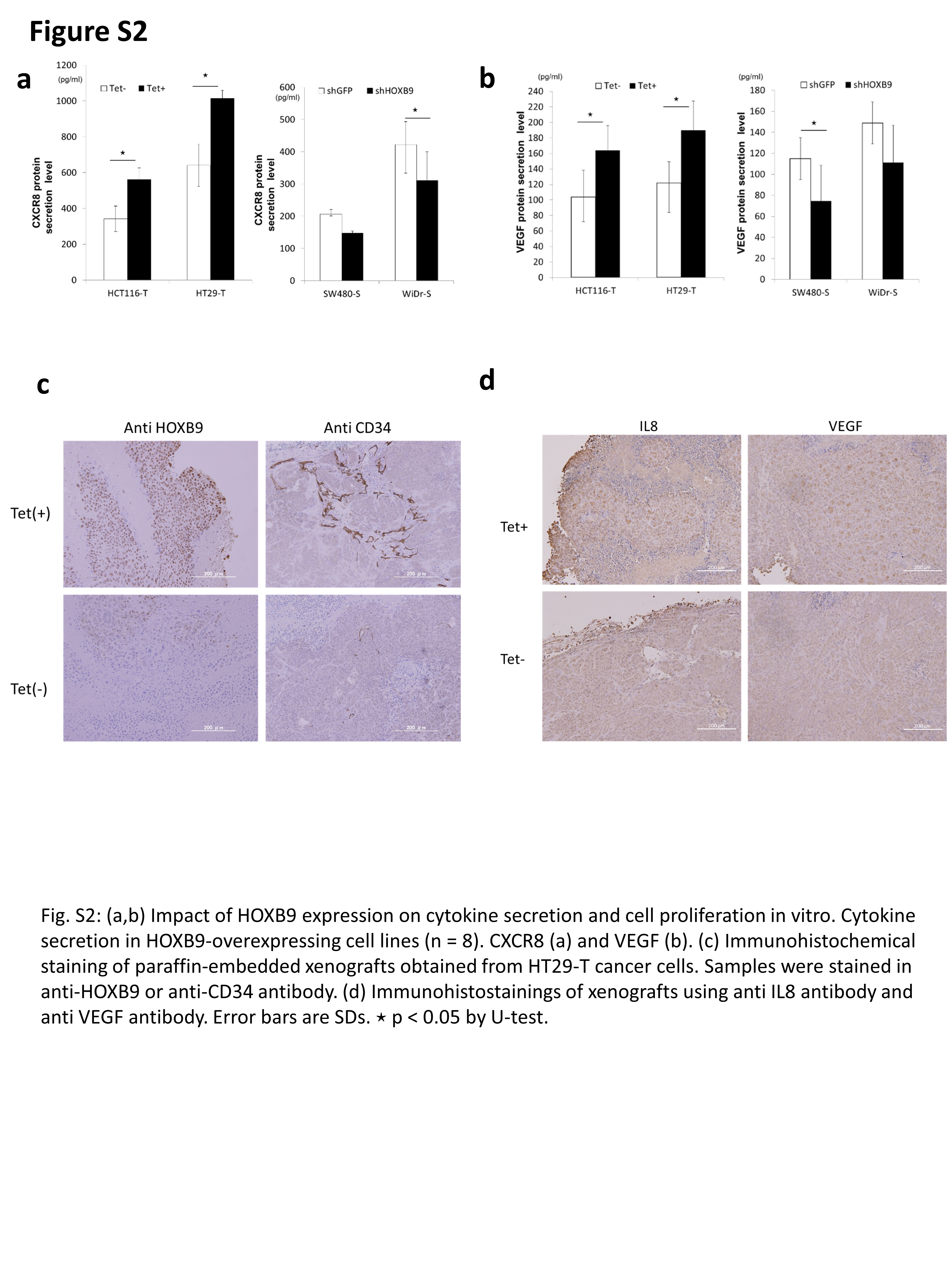

Supplement: Additional file 2: Figure S2 — (a, b) Impact of HOXB9 expression on cytokine and cell proliferation in vitro. Cytokine secretion in HOXB9-over expressing cell lines (n=8). CXCR8 (a) and VEGF (b). (c) Immunohistochemical staining of paraffin-embeddedxenografts obtain from HT29-T cancer cells. Samples were stained in anti-HOXB9 or anti-CD34 antibody. (d) Immunohistostaining of xenografts using anti IL8 antibody and anti VEGF antibody. Error bars are SDs. * p <0.05 by U-test. [file 1476-4598-13-102-S2.tiff]

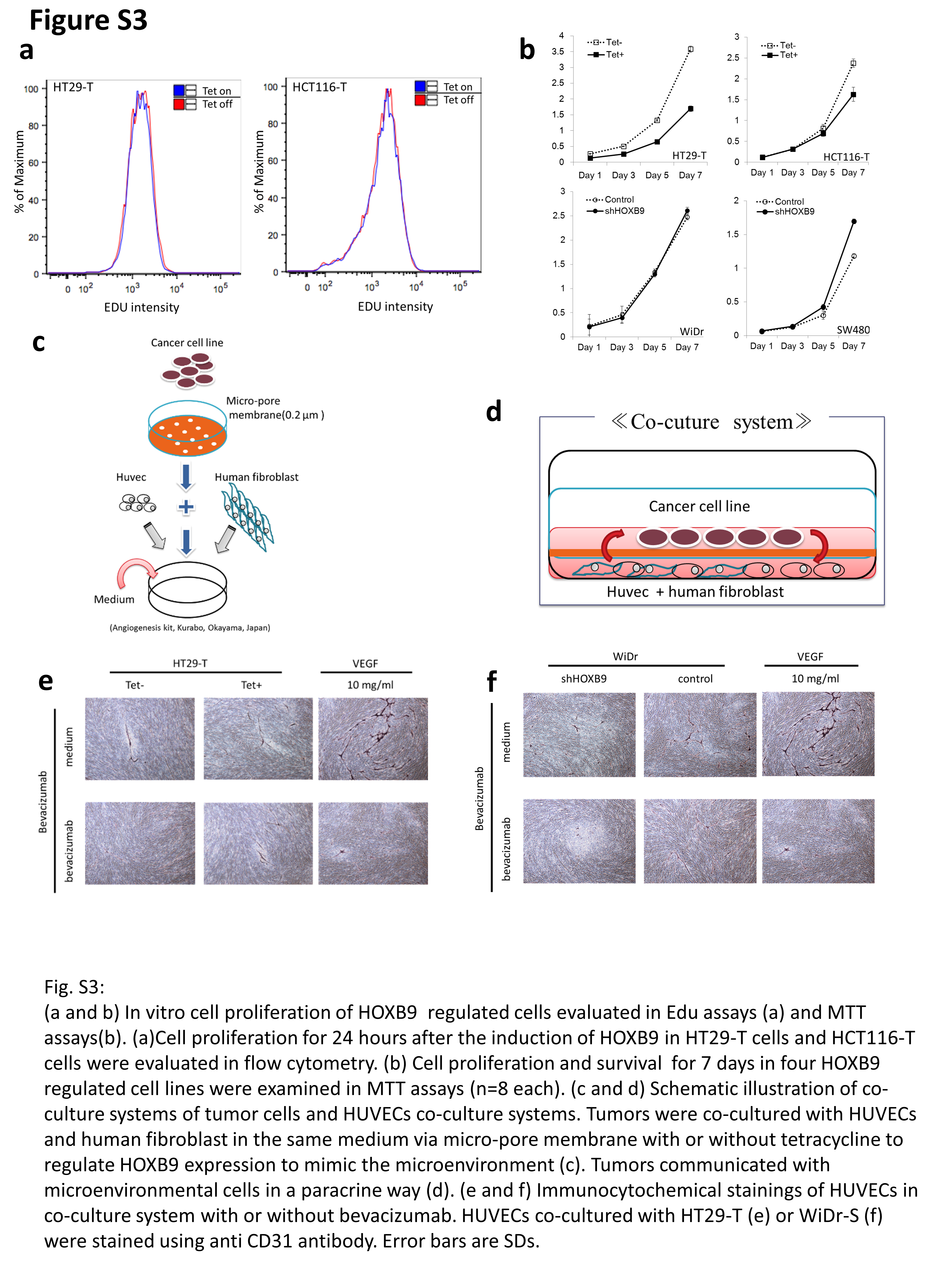

Supplement: Additional file 3: Figure S3 — (a and b) In vitro cell proliferation of HOXB9 regulated cells evaluated in Edu assays (a) and MU assays (b). (a) Cell proliferation for 24 hours after the induction of HOXB9 in HT29-T cells and HCT116-T cells were evaluated in flow cytometry. (b) Cell proliferation and survival for 7 days in four HOXB9 regulated cell lines were examined in MU assays (n=8 each). (c and d) Schematic illustration of coculture systems of tumor cells and HUVECs co-culture systems. Tumors were co-cultured with HUVECs and human fibroblast in the same medium via micro-pore membrane with or without tetracycline to regulate HOXB9 expression to mimic the microenvironment (c). Tumors communicated with microenvironmental cells in a paracrine way (d). (e and f) Immunocytochemical stainings of HUVECs in co-culture system with or without bevacizumab. HUVECs co-cultured with HT29-T (e) or WiDr-S (f) were stained using anti CD31 antibody. Error bars are SDs. [file 1476-4598-13-102-S3.tiff]

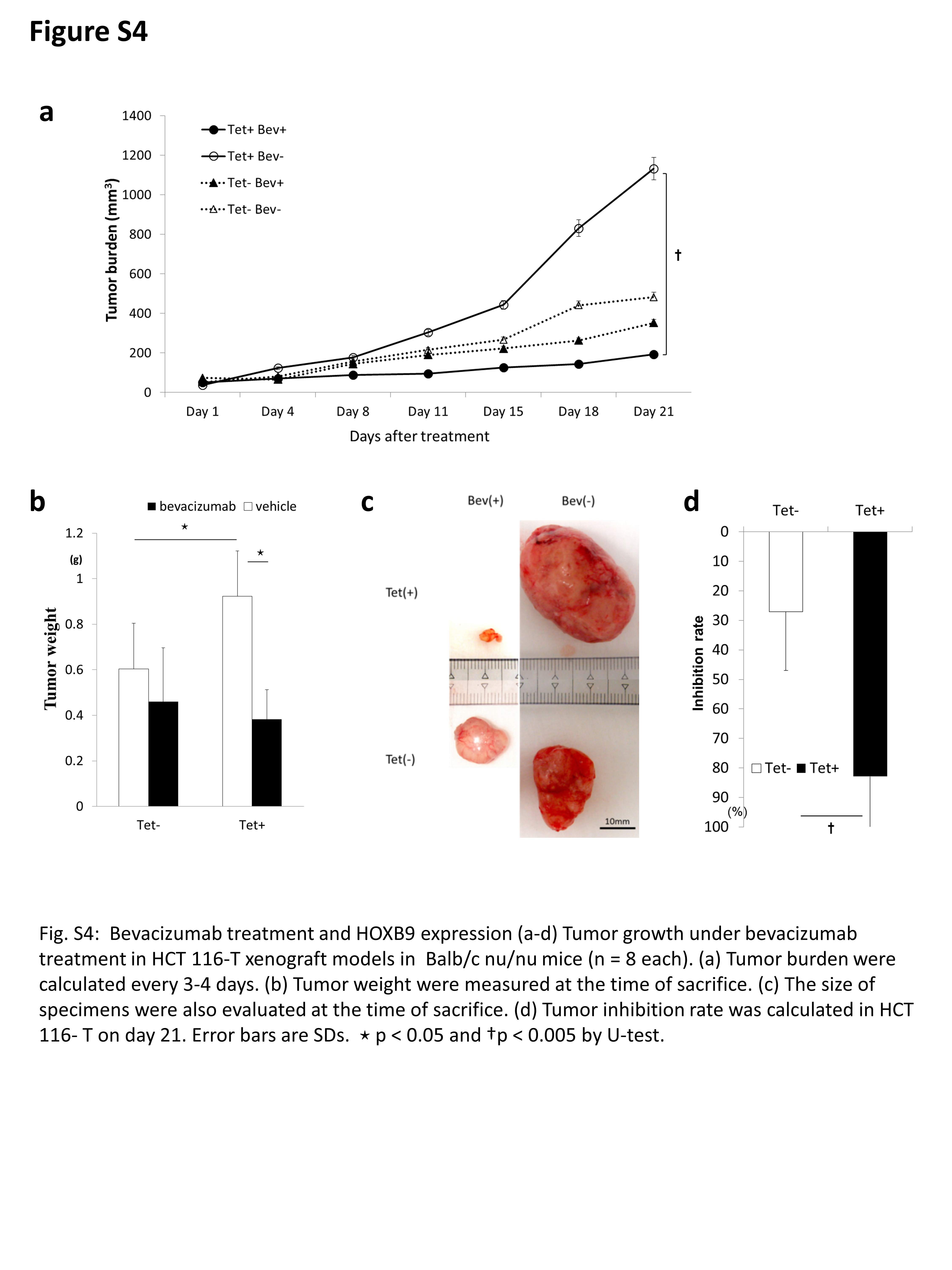

Supplement: Additional file 4: Figure S4 — Bevacizumab treatment and HOXB9 expression (a-d) Tumor growth under bevacizumab treatment in HCT 116-T xenograft models in Balb/c flu/flu mice (n = 8 each). (a) Tumor burden were calculated every 3-4 days. (b) Tumor weight were measured at the time of sacrifice. (c) The size of specimens were also evaluated at the time of sacrifice. (d) Tumor inhibition rate was calculated in Ha 116- T on day 21. Error bars are SDs. *p < 0.05 and tp < 0.005 by U-test. [file 1476-4598-13-102-S4.tiff]

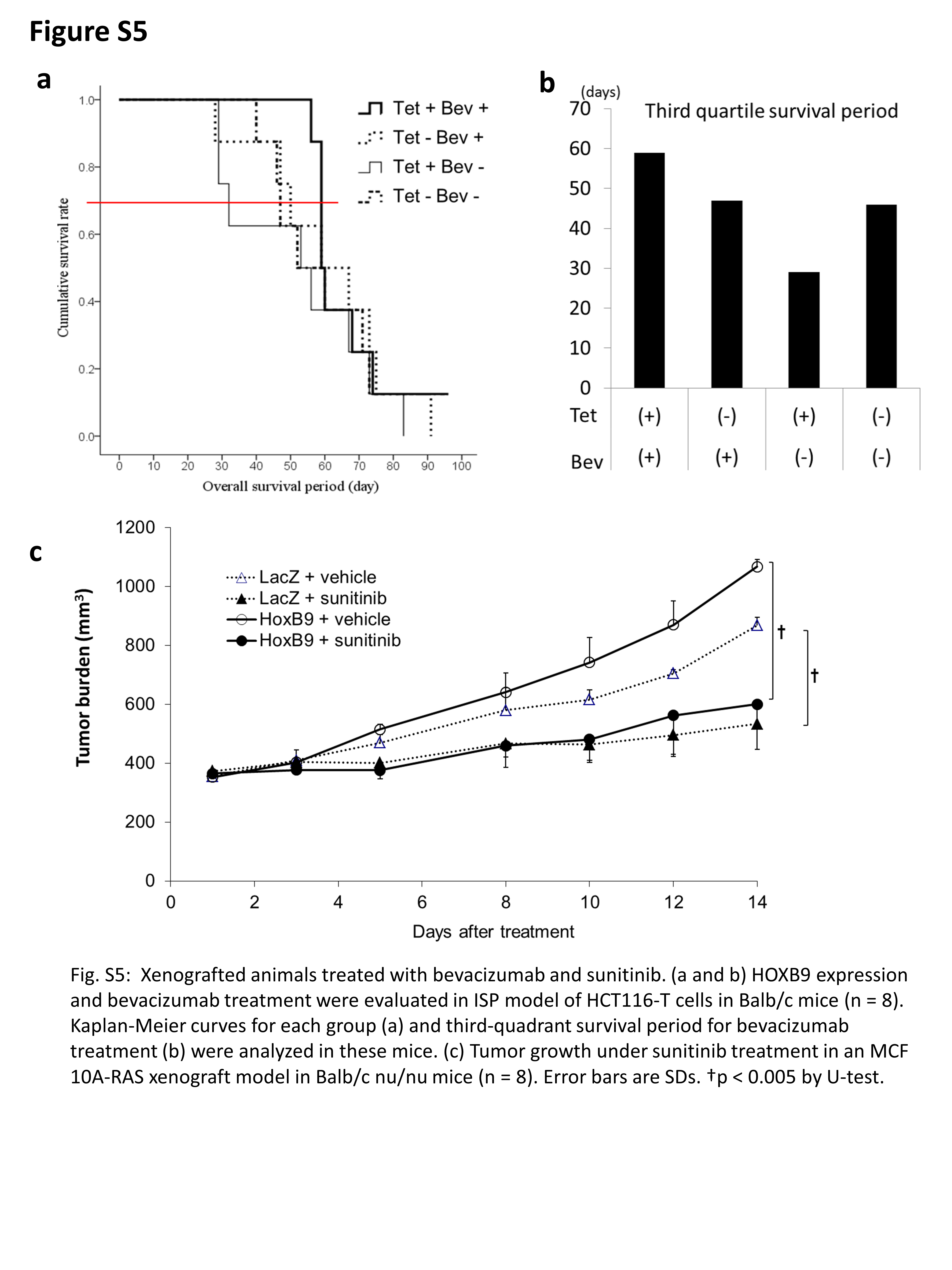

Supplement: Additional file 5: Figure S5 — Xenografted animals treated with bevacizumab and sunitinib. (a and b) HOXB9 expression and bevacizumab treatment were evaluated in ISP model of HCT116-T cells in Balb/c mice (n = 8). Kaplan-Meier curves for each group (a) and third-quadrant survival period for bevacizumab treatment (b) were analyzed in these mice. (c) Tumor growth under sunitinib treatment in an MCF 1OA-RAS xenograft model in Balb/c nu/nu mice (n = 8). Error bars are SDs. tp < 0.005 by U-test. [file 1476-4598-13-102-S5.tiff]

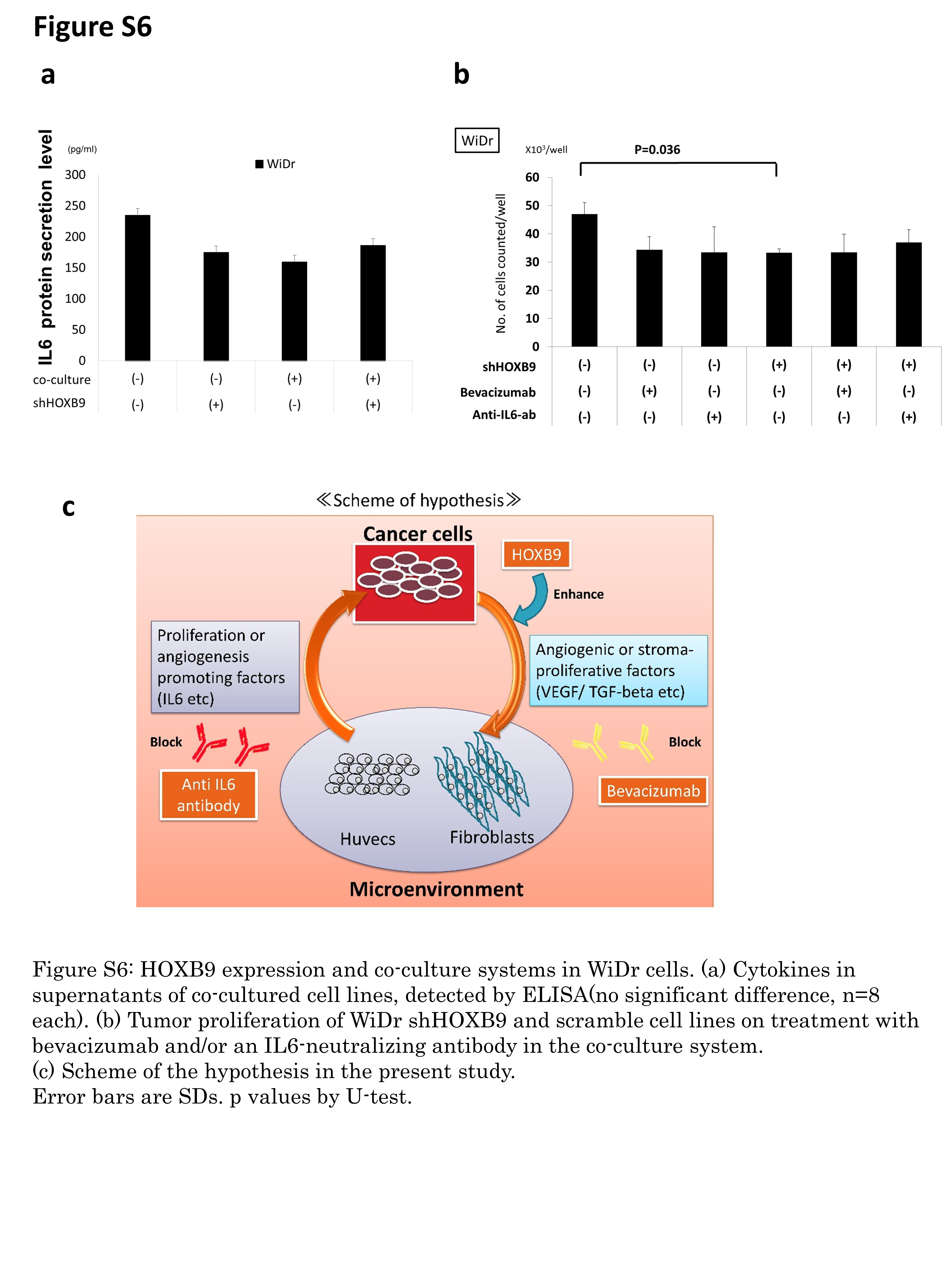

Supplement: Additional file 6: Figure S6 — HOXB9 expression and co-culture systems in WiDr cells. (a) Cytokines in supernatants of co-cultured cell lines, detected by ELISA (no significant difference, n8 each). (b) Tumor proliferation of WiDr shHOXB9 and scramble cell lines on treatment with bevacizumab andlor an 1L6-neutralizing antibody in the co-culture system. (c) Scheme of the hypothesis in the present study. Error bars are SDs. p values by U-test. [file 1476-4598-13-102-S6.tiff]

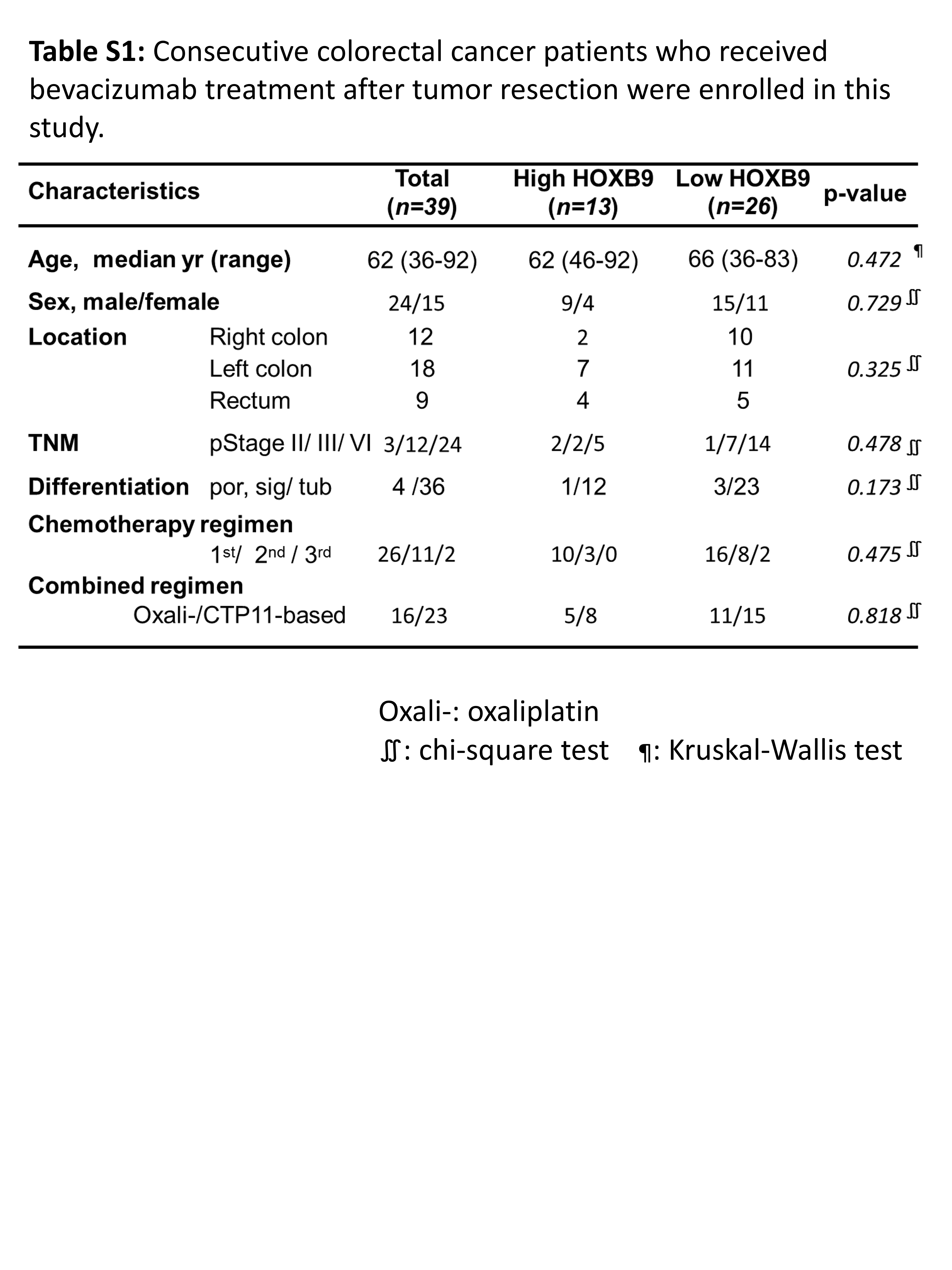

Supplement: Additional file 7: Table S1 — Consecutive colorectal cancer patients who received bevacizumab treatment after tumor resection were enrolled in this study. [file 1476-4598-13-102-S7.tiff]

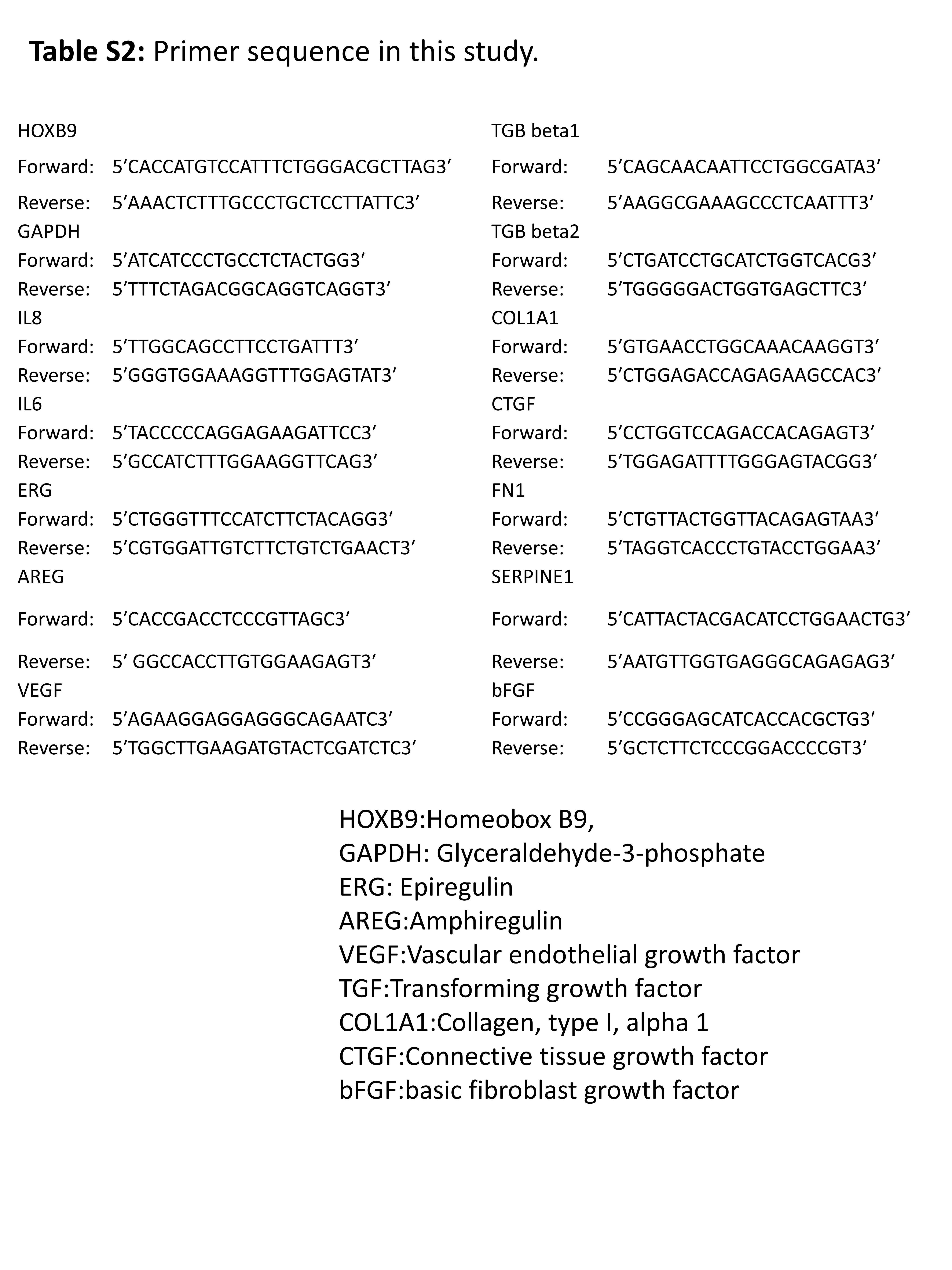

Supplement: Additional file 8: Table S2 — Primer sequence in this study. [file 1476-4598-13-102-S8.tiff]
